# YueF Overexpression Inhibits Cell Proliferation Partly through p21^WAF1/Cip1^ Upregulation in Renal Cell Carcinoma

**DOI:** 10.3390/ijms12042477

**Published:** 2011-04-11

**Authors:** Hsuan-Wei Huang, Jian-Ping Peng, Jie Zhang

**Affiliations:** 1 Department of Urology, RenMin Hospital of Wuhan University, Wuhan 430060, China; E-Mails: xuanwei_huang@163.com (H.-W.H.); jianping0209@163.com (J.-P.P.); 2 State Key Laboratory of Virology, Wuhan University, Wuhan 430071, China

**Keywords:** YueF, clear cell RCC, p53, p21

## Abstract

YueF is a novel putative tumor suppressor gene that can inhibit proliferation and induce apoptosis in hepatoma cells, but its role in renal cell carcinoma (RCC) remains unclear. Here, we examined the expression of the YueF gene in RCC tissues and the effect of YueF on cell proliferation in RCC 786-0 cells. The results showed that YueF was expressed at high levels in normal kidney tissues and cell lines but was reduced or absent in RCC tissues and 786-0 cells. Lentivirus-mediated YueF overexpression in RCC 786-0 cells caused cell-cycle arrest in the G1 phase and dramatically reduced proliferation in culture. YueF overexpression resulted in increased protein levels of p53 and p21^WAF1/Cip1^, whereas the protein levels of cyclin D1 and pRb were decreased. The proliferation defects caused by YueF overexpression could be partially rescued by the expression of p21 siRNA. These findings suggest a critical role for p21 in the YueF-induced growth inhibition of 786-0 cells and provide novel insights into the mechanism underlying the tumor-suppressive action of YueF.

## 1. Introduction

Renal cell carcinoma (RCC) is the most common type of kidney cancer and follows an unpredictable disease course [1]; it is the most malignant tumor of the kidney and accounts for approximately 2–3% of all adult cancers in Western countries. Moreover, the morbidity and mortality associated with renal carcinoma has steadily increased over the past 20 years. Histopathologically, RCC is classified into four subtypes, with 80% of cases consisting of the clear cell subtype [2]. Due to its insidious onset, patients frequently exhibit advanced disease at the time of clinical diagnosis; drugs and newly discovered markers have been unable to significantly increase the survival of these patients [3]. Therefore, additional research is required to identify specific genes that might play important roles in RCC carcinogenesis.

YueF (GenBank accession number: BC006131) is a putative tumor suppressor gene that is expressed in hepatomas [4]. It was initially discovered by Huang *et al.* [4,5] using a yeast two-hybrid method aimed at identifying Hepatitis B virus X protein (HBx)-interacting proteins. YueF is highly expressed in the cytoplasm of normal cells and tissues, including liver, lung, bladder, myocardial tissue, and intestine; however, it is detected at low levels in corresponding tumor tissues, including liver, lung, and bladder cancers. Overexpression of YueF can inhibit the proliferation of hepatocellular carcinoma (HCC) cells, induce apoptosis *in vitro* and suppress the HCC tumorigenicity in nude mice *in vivo*, indicating that YueF might be a novel candidate gene for tumor suppression in tumorigenesis. However, the biological role and the mechanism of YueF action in renal cell carcinoma (RCC) are largely unknown.

We investigated not only YueF expression in RCC tissues and corresponding normal tissues but also studied the role of YueF in the proliferation of RCC cells. Our data indicated that YueF expression decreased in RCC tissues and 786-0 cells. YueF overexpression resulted in increased levels of p53 and p21^WAF1/Cip1^ protein expression and a decreased level of cyclin D1 expression and subsequently induced inhibition of cell growth in 786-0 cells. The proliferation defects caused by YueF overexpression can be partially rescued with p21 siRNA. This study is the first to demonstrate that YueF modulates cell growth via the regulation of the p21^WAF1/Cip1^ pathway in renal cell carcinoma.

## 2. Materials and Methods

### 2.1. Human Tissue Preparation

Tissue samples from eight human renal cell carcinomas and adjacent normal tissues were obtained after nephrectomy in RCC patients (2 females and 6 males) and examined by a pathologist. All the eight RCC samples are clear cell subtype, and all the adjacent tissue samples are normal, without fibrosis or other non-neoplastic changes. Four tumors were in stage I, three in stage II and one in stage III. All tissue samples were immediately dissected, frozen in liquid nitrogen and stored at −80 °C. Tissue samples (1 g) were washed with normal saline (NS) to remove cell debris and any remaining blood before trituration with liquefacient nitrogen and subsequent homogenization in lysis buffer (9 M Urea, 4% CHAPS, 65 mM DTT, Rose’s enzyme inhibitor cocktail); after being completely dissolved by ultrasonic fragmentation, the homogenates were centrifuged at 100,000 g for 10 min at 4 °C. The supernatants were kept in small aliquots at −80 °C. The total protein concentration was determined with the BCA method.

### 2.2. Generation of Recombinant Lentivirus

Total RNA was isolated from normal human tissue using TRIzol TM Reagent (GIBCO, Invitrogen); RT-PCR was performed using a First Strand cDNA Synthesis Kit (MBI, Fermentas, Lithuania). The YueF gene was amplified with PCR (Takara, Tokyo, Japan). The following specific primers were use: sense primer 5′ TTGTCTAGAGCCACCATGGCTGCAAGTGGCCGAGGTCTCT 3′ and anti-sense primer 5′ TATGCGGCCGCTCACATGCTCTTGAGGTCCCTAAAG 3′, which contained Xbal and Not1 sites (underlined), respectively. The PCR product was subcloned into the lentiviral vector pCDH-CMV-MCS-EF1-copGFP (System Biosciences, Mountain View, CA, USA) to generate pCDH-YueF. Then, the lentiviral YueF plasmid was co-transfected into 293FT cells (Invitrogen, USA) along with three different packaging plasmids, pLP1, pLP2, and pLP/VSV-G, to generate lentivirus [6].

### 2.3. Cell Culture and Lentivirus Transduction

Human RCC 786-0 cells were cultured in Eagle’s MEM supplemented with 10% foetal bovine serum (FBS) (Hyclone, Logan, UT, USA) at 37 °C under 5% CO_2_. The 786-0 cells were infected at a multiplicity of infection (MOI) of 5 with either lentivirus containing the YueF gene (pCDH-YueF) or empty virus (EV) containing 6 μg/mL of polybrene according to the manufacturer’s instructions. RT-PCR was used to examine gene expression in the 786-0 cells infected with pCDH-YueF. The following primers were used in the RT-PCR: YueF sense primer 5′-GGCGTGGAGACGAGATAA-3′, YueF anti-sense primer 5′-GAGGGTCAATGGCTAATGC-3′, β-actin sense primer 5′-TCCCTGGAGAAGAGCTACGA-3′, and β-actin anti-sense primer 5′-TCGTCATACTCCTGCTTGCT-3′.

### 2.4. Cell Proliferation Assay

To examine the effect of YueF on cell growth, 786-0 cells were infected with either lentivirus containing the YueF gene (pCDH-YueF) or EV. The infected cells were seeded into 96-well plates and incubated for 1 to 6 days. Subsequently, 20 μL of a 3-(4,5-dimethylthiazol-2-yl)-2,5-diphenyl tetrazolium bromide (MTT, Sigma) solution (5 mg/mL, Sigma) was added to each well, and the plates were further incubated for 3 h. Crystals were dissolved in 0.04 M HCl in isopropanol. The absorbance at 490 nm was measured with a microplate reader (Bio Rad, Hercules, CA, USA). The experiments were independently repeated 3 times.

### 2.5. p21 siRNA Transfection

Transfecting cells with siRNA specific for human p21 achieved suppression of p21 in 786-0 cells. Control siRNA and p21 siRNA (and a pool of 3–4 individual siRNAs targeting different regions of p21 mRNA) are commercially available from Ambion. siRNA transfection was performed at a working concentration of 25 nM using a transfection reagent according to the manufacturer’s instructions. All experiments were independently performed at least 3 times, and similar results were obtained [7].

### 2.6. Western Blot Analysis

Cell extracts were obtained using lysis buffer containing 5 mmol/L EDTA, 1 mmol/L phenylmethylsulfonyl fluoride, 1 mmol/L dithiothreitol, 0.1 mmol/L leupeptin, 75 μmol/L pepstatin A, 150 mmol/L NaCl, and 0.1% Triton X-100. The mixture was centrifuged for 30 min (15,000 rpm, 4 °C), and the supernatant was collected for Western blot analysis. Samples (20 μg) were electroblotted onto nitrocellulose membranes. An anti-YueF polyclonal antibody (Mr. Wu, State Key Laboratory of Virology, Wuhan University, China), and anti-human p53, p21, cyclin D1, CDK2, pRb (Ser780), and beta-actin polyclonal antibodies (Cell Signaling) were used as the primary antibodies; peroxidase-conjugated goat anti-rabbit or anti-mouse antibodies (Pierce) were used as the secondary antibodies. Protein bands were visualized using the SuperSignal West Pico Chemiluminescent Substrate (Pierce).

### 2.7. Cell Cycle Analysis

Cells grown in regular growth or serum-free media for 36 h were collected, fixed in methanol, and stained with PBS containing 10 μg/mL propidium iodide and 0.5 mg/mL RNase A for 15 min at 37 °C. The DNA content of the labeled cells was measured using the FACSCalibur flow cytometry system (BD Biosciences). Each experiment was performed in triplicate.

### 2.8. Statistical Analysis

Experiments were repeated three times and the results were expressed as the mean ± SD. The Student’s t-test or ANOVA followed by a post-hoc test was used to compare the values of the tumor samples to those of the control samples. A value of *P* < 0.05 was considered to be statistically significant.

## 3. Results

### 3.1. Downregulated Expression of YueF Protein in Clinical RCC Tissues and RCC 786-0 Cells

**Figure 1.**
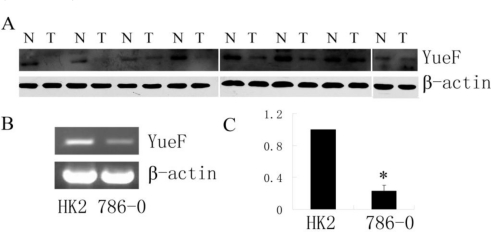
Expression of YueF in RCC tissues and 786-0 cells. (**A**) Western blot showing YueF expression in 8 human RCC tissues (T) and corresponding normal samples (N), results were from 3 different gels. (**B**) (**C**) RT-PCR for YueF mRNA expression in human proximal tubule epithelial HK-2 cells and RCC 786-0 cells. β-actin was used as the internal control (* *P* < 0.05).

Western blot analysis was performed to detect YueF protein expression in RCC tissues. As shown in Figure 1, the expression of YueF was observed in the normal samples but was decreased by 10–95% of the normal tissue levels in the corresponding RCC tissue samples from 7 of the 8 tumors; however, no expression of YueF was observed in one of the RCC tumor samples (Figure 1A). It was also observed that the levels of YueF mRNA were high in the normal human renal proximal tubular cell line, HK2, but the expression levels were reduced in the RCC cell line, 786-0 (Figure 1B, C). These results demonstrated that although YueF was highly expressed in normal tissues and cells, its expression was decreased in RCC tissues and cells.

### 3.2. Overexpression of YueF in RCC 786-0 Cells with Lentivirus

**Figure 2.**
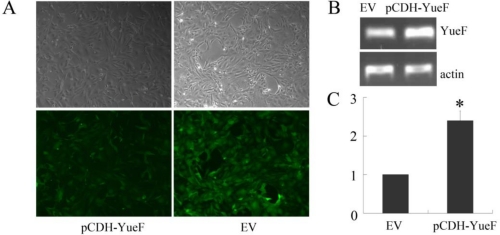
Restored expression of YueF in RCC 786-0 cells. (**A**) Infection efficiency in RCC 786-0 cells. (**B**, **C**) Results of the RT-PCR assay show a significantly increased mRNA level of YueF in YueF-overexpressing 786-0 cells (pCDH-YueF) compared with empty virus-infected 786-0 cells (EV). β-actin was used as the internal control (* *P* < 0.05).

To study the biological functions of YueF, we introduced YueF into 786-0 RCC cells using a lentivirus containing the YueF gene. The 786-0 cells were infected with either virus containing the YueF gene (pCDH-YueF) or an empty virus (EV); RT-PCR was used to identify YueF mRNA expression after infection. As shown in Figure 2, the infection efficiency was approximately 100% in the 786-0 cells (Figure 2A), and the YueF mRNA level was significantly higher in YueF-overexpressing 786-0 cells (pCDH-YueF) compared to the empty virus (EV)-infected control 786-0 cells (Figure 2B and C).

### 3.3. YueF Overexpression Inhibits the Proliferation of 786-0 Cells

**Figure 3.**
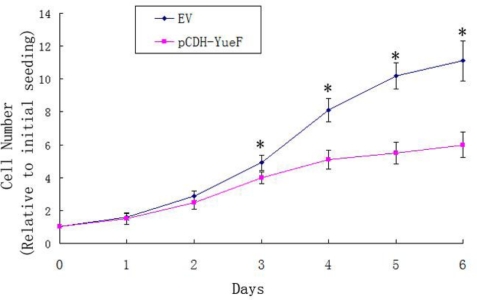
Inhibition of cell proliferation in YueF-overexpressing 786-0 cells (pCDH-YueF) compared with empty virus-infected 786-0 cells (EV) (*P < 0.05).

The effects of YueF overexpression on the proliferation 786-0 cells were examined. The growth curve results determined with an MTT assay showed that the overexpression of YueF (pCDH-YueF) significantly reduced the proliferation rate compared to the empty virus (EV)-infected cells (*P* < 0.05, Figure 3).

### 3.4. YueF Overexpression in 786-0 Cells Causes Cell Cycle Arrest at the G1 Phase

**Figure 4.**
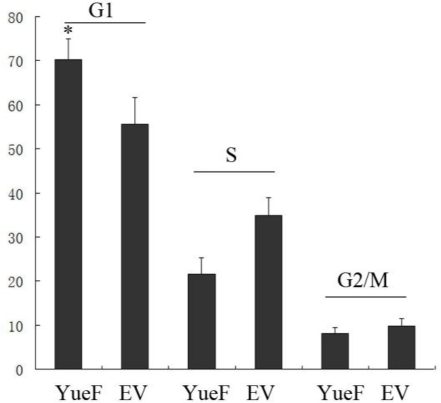
Cell cycle arrest at the G1 phase was induced in the YueF-overexpressing 786-0 cells (YueF) compared to the empty virus (EV)-infected 786-0 cells. (*P < 0.05)

Huang *et al.* [5] showed that overexpression of YueF inhibited hepatoma cell proliferation through the induction of apoptosis. Our results did not show apoptotic induction in the YueF-overexpressing 786-0 cells (pCDH-YueF); therefore, we determined the cell cycle distribution. The S-phase population was markedly decreased, and the G1 population was significantly increased in the YueF-overexpressing 786-0 cells (YueF) compared to the empty virus-infected 786-0 cells (EV) (*P* < 0.05). Neither YueF nor EV cells exhibited significant changes in the G2 population (Figure 4).

### 3.5. YueF Overexpression Inhibits the Proliferation of 786-0 Cells Partly through p21 Upregulation

**Figure 5.**
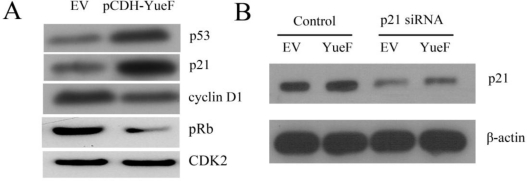

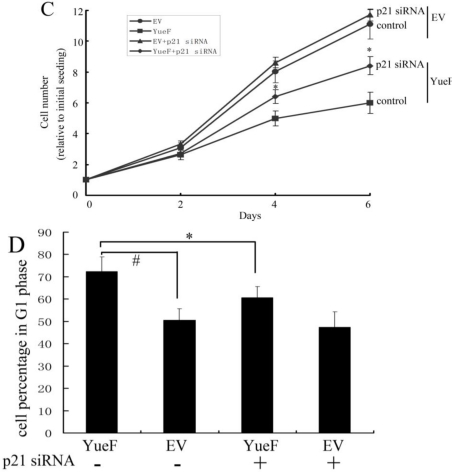
YueF overexpression partially inhibits the proliferation of 786-0 cells through p21 upregulation. (**A**) YueF overexpression results in upregulation of p53 and p21 levels, but downregulation of cyclin D1 and pRb levels in RCC 786-0 cells; (**B**) Western blot analysis for p21 protein demonstrates strong suppression of p21 expression after treatment with 25 nM of p21 siRNA; (**C**) Knockdown of p21 expression in YueF-overexpressing (YueF) and empty virus (EV)-infected 786-0 cells led to a substantially faster growth rate in the YueF-overexpressing 786-0 cells; (**D**) Silencing p21 expression partially restored the G1 arrest induced by YueF overexpression. (*P < 0.05 p21 siRNA *vs.* scramble control; ^#^*P* < 0.05 YueF *vs.* EV)

Huang *et al.* showed [5] that YueF overexpression enhanced p53 promoter activity and p53 expression in hepatoma cells. Here, we also showed that p53 expression increased in YueF-overexpressing cells (Figure 5A). To further explore how YueF overexpression induced cell cycle arrest, we examined the relative expression levels of p21 (a major cyclin-dependent kinase inhibitor) in YueF-overexpressing 786-0 cells (pCDH-YueF) and empty virus-infected 786-0 cells (EV) with immunoblot analysis, which showed a dramatic elevation in p21 levels in the YueF-overexpressing 786-0 cells (Figure 5A). Consistent with the G1-arrest phenotype, the YueF-overexpressing cells (pCDH-YueF) showed substantially lower cyclin-D1 and pRB (Ser780) levels compared to the control cells (EV), whereas there was no change in the CDK2 protein levels (Figure 5A).

To determine whether an elevated p21 level is at least partially responsible for the YueF-induced growth suppression of 786-0 cells, we silenced p21 expression in YueF-overexpressing 786-0 cells (pCDH-YueF) and empty virus (EV)-infected 786-0 cells with transient transfection of a pool of p21-specific siRNAs and scrambled control siRNA. Western blot data (Figure 5B) confirmed that p21 expression could be suppressed by p21 siRNA treatment. In contrast, silencing p21 expression led to substantially faster growth of the YueF-overexpressing 786-0 cells (Figure 5C). The YueF-overexpressing 786-0 cell counts remained lower than the corresponding values for the EV-infected cells, suggesting that the p21 upregulation is only partly responsible for the growth inhibition of 786-0 cells induced by YueF overexpression. Additionally, silencing p21 expression rescued the G1 arrest induced by YueF overexpression to some extent (Figure 5D).

## 4. Discussion

Although the tumor-suppressive action of YueF in hepatoma cells has been previously reported [5], the biological role of YueF and the molecular pathways through which YueF upregulation suppresses the growth of renal cell carcinoma (RCC) were not elucidated in earlier studies. YueF is expressed at high levels in normal human hepatic cells and tissues, but barely detectable in hepatoma cells and tissues. Overexpression of YueF could inhibit cell proliferation, induce cell apoptosis and promote p53 expression in hepatoma cells, indicating that YueF might be a novel candidate tumor suppressor gene involved in hepatocarcinogenesis.

This study demonstrated that the YueF protein was highly expressed in normal kidney tissues but absent or decreased in RCC tissues. It was also expressed at high levels in the normal human renal proximal tubular cell line HK2 but was decreased in RCC 786-0 cells. YueF overexpression led to increased protein levels of p53 and p21^WAF1/Cip1^; the levels of cyclin D1 and pRb were decreased, resulting in reduced cell growth and G1/S cell-cycle arrest *in vitro*. The proliferation defects caused by YueF overexpression could be partially rescued by p21 siRNA expression, suggesting that factors in addition to p21 upregulation contribute to the decreased proliferation of YueF-overexpressing cells (we also observed a significant decrease in the cyclin D1 level in YueF-overexpressing 786-0 cells).

Expression of YueF activated p53 expression in both HCC and RCC cells; apoptosis was induced in HCC cells, while G1/S cell-cycle arrest was induced in RCC 786-0 cells. Activation of p53 expression can trigger various cellular responses that can lead to cell-cycle arrest, senescence, differentiation, DNA repair, apoptosis, and inhibition of angiogenesis. Furthermore, p53-dependent transcriptional regulation of p21, Cdc25C, and GADD45 has been proposed to mediate cell growth arrest [8–11], and the regulation of the Bcl-2 family of proteins by p53 mediates the mitochondrial pathway of apoptosis [12]. However, the mechanisms underlying p53-activated cell growth arrest and apoptosis are poorly understood. It has been proposed that this process may be driven by affinity [13]. Low levels of stress or DNA damage could induce a low level of p53 expression that causes high-affinity growth arrest genes to be upregulated. In contrast, when stress levels are high, induction of p53 could be further increased to activate apoptotic genes. Another possibility is that RCC 786-0 cells are von Hippel-Lindau (VHL) deficient. The expression levels of the pro-apoptotic Bcl-2 family protein, BIM-EL, in these cells is low, and therefore, RCC 786-0 cells are more resistant to certain apoptotic stimuli than the same cells that stably express the wild-type VHL protein [14,15].

The potent cyclin-dependent kinase (CDK) inhibitor, p21 [16], binds to and inhibits the activity of CDK4/6-cyclin D complexes. The expression of p21 is strictly controlled by the tumor suppressor protein p53, which mediates the p53-dependent G1 phase of the cell cycle [17]. The retinoblastoma tumor suppressor gene (pRb) is a substrate of CDK4/6-cyclin D. When a cell prepares to enter S phase, complexes of CDK4/6-cyclin D phosphorylate pRb and inhibit its activity; E2F transcription factors [18,19] are positively regulated and induce S phase entry. The phosphorylation of pRb is inhibited by p21, resulting in the negative regulation of E2F transcription factors and cell-cycle G1 phase arrest. In the present study, it was found that YueF stimulates p53 transcriptional activity and mediates G1 cell-cycle arrest through the upregulation of p21, repression of cyclin D1, and dephosphorylation of pRb.

Here, we observed that the level of pRb, which is a CDK substrate, was decreased in YueF-overexpressing cells, whereas CDK2 levels did not show any change. Generally, p21 not only inhibits the activity of CDK4/6-cyclin D complexes and induces G1 phase arrest but also inhibits cyclin E/CDK2 complexes and entry into S phase. CDK protein levels always remain stable, whereas the levels of cyclins fluctuate periodically during the cell cycle [20]. CDK2 activity is not associated with CDK2 protein levels. There are at least four major mechanisms involved in the inactivation of CDK2 kinase: dephosphorylation of Thr160, phosphorylation of Thr14 and Tyr15, removal of cyclin E, and binding of cyclin-dependent kinase inhibitors (CKI) [21]. Some reports have also shown that CDK2 activity can be decreased without suppressing CDK2 protein levels [22,23].

The mechanism by which YueF overexpression activates p53 remains unknown. Huang *et al*. showed [5] that YueF overexpression enhanced p53 promoter activity in HepG2 hepatoma cells. YueF was initially identified as a Hepatitis B virus X protein (HBx)-interacting protein. HBx inhibits p53 promoter activity and protein expression, while YueF overrides the function of HBx by promoting p53 protein expression in hepatoma cells, suggesting that YueF is a strong antagonist of the p53 negative regulator. Murine double minute 2 (MDM2), a key negative regulator of p53 stability and activity, can bind to p53 and block p53 transcriptional activity [24]. YueF may also interact with MDM2 and upregulate p53 expression; however, this hypothesis requires further investigation.

## 5. Conclusion

Here, we report the first investigation of how YueF upregulation alters the cell-cycle progression of 786-0 cells. We observed the downregulation of YueF in RCC tissues and 786-0 cells. We further present the novel findings that YueF overexpression in 786-0 cells is associated with a dramatic upregulation of p21 levels, cell-cycle arrest at the G1 phase, and reduced cell proliferation *in vitro*.
